# Rapid Atrial Pacing After TAVI for Pacemaker Prediction

**DOI:** 10.31083/RCM39074

**Published:** 2025-08-15

**Authors:** Leonidas Koliastasis, Ioannis Doundoulakis, Dimitrios Tsiachris, Luigi Pannone, Ioannis Skalidis, Quentin de Hemptinne, Panagiotis Xaplanteris, Konstantinos Toutouzas, Carlo de Asmundis

**Affiliations:** ^1^Department of Cardiology, Université Libre de Bruxelles (ULB), Centre Hospitalier Universitaire Saint-Pierre, 1000 Brussels, Belgium; ^2^Unit of Structural Heart Disease and Valvular Disease, First Department of Cardiology, National and Kapodistrian University of Athens, Hippokration Hospital, 11527 Athens, Greece; ^3^Heart Rhythm Management Centre, Postgraduate Program in Cardiac Electrophysiology and Pacing, Universitair Ziekenhuis Brussel - Vrije Universiteit Brussel, European Reference Networks Guard-Heart, 1090 Brussels, Belgium; ^4^Service de Cardiologie, Institut Cardiovasculaire Paris Sud, 91300 Massy, France

**Keywords:** pacemaker, predictors, rapid atrial pacing, TAVI, TAVR

## Abstract

Despite continued advancements in transcatheter aortic valve implantation (TAVI) techniques, the incidence of permanent pacemaker implantation (PPI) remains substantial. Established predictors of PPI include advanced age, pre-existing electrocardiographic conduction abnormalities, prosthetic valve type, implantation depth, and anatomical parameters, such as membranous septum length, which are currently under active investigation. In routine clinical practice, the management strategy often involves the temporary placement of a transvenous pacemaker lead, followed by a period of observation. While widely implemented, this approach introduces clinical uncertainty and may contribute to prolonged hospitalization, particularly given the not infrequent occurrence of delayed high-degree atrioventricular (AV) block. A novel diagnostic method emerging from electrophysiological evaluation is rapid atrial pacing performed post-TAVI, which aims to assess susceptibility to Wenckebach-type AV block. Two observational studies have evaluated this technique, utilizing an upper pacing threshold of 120 beats per minute as a cutoff to identify patients at risk of requiring permanent pacing. Moreover, this method is cost-effective, technically straightforward, and time-efficient; preliminary findings suggest this technique possesses a high negative predictive value. However, additional prospective data are required to validate the clinical utility of this technique and inform the development of standardized implementation. An upcoming clinical study (NCT06189976) is anticipated to provide valuable insights.

## 1. Introduction

Despite considerable advancements in transcatheter aortic valve implantation (TAVI), including refinements in procedural techniques, device technology, and operator expertise, the requirement for permanent pacemaker implantation (PPI) following the procedure remains a significant limitation. According to data from the CENTER collaboration, PPI rates demonstrate substantial variability, ranging from 7.8% to 20.3%, with notable differences observed across commercially available valve platforms [1]. While PPI contributes to extended hospital stays and increased healthcare expenditures, emerging evidence suggests that its implications may extend beyond the perioperative period. A recent meta-analysis reported an association between post-TAVI PPI and increased all-cause mortality, as well as higher rates of rehospitalization during long-term follow-up [2]. Conversely, findings from the SWEDEHEART registry indicated no significant difference in survival at a median follow-up of 2.7 years [3]. Nevertheless, as TAVI is increasingly offered to younger and lower-risk patient populations, the potential long-term consequences of PPI necessitate further rigorous investigation. In support of evolving practice trends, a large-scale registry of over 50,000 TAVI procedures using the self-expanding Evolut (Medtronic, Minneapolis, MN, USA) platform demonstrated a decline in both in-hospital (8.8%) and 30-day (10.8%) PPI rates [4]. Despite those encouraging data, heterogeneity persists across studies. For instance, the recent LANDMARK randomized controlled trial reported a 30-day PPI incidence of 15% following implantation with the Myval (Meril Life Sciences, Vapi, Gujarat, India) balloon-expandable valve, underscoring the need for ongoing evaluation of device-specific outcomes [5].

## 2. Opinion

Clinicians must remain vigilant for signs indicative of the need for PPI following TAVI, as the delayed onset of high-degree atrioventricular (AV) block is not uncommon. Predictive factors for PPI have been extensively investigated, with male sex and baseline electrocardiographic (ECG) conduction disturbances (CDs) identified as principal determinants [6]. Both pre- and post-procedural ECGs may serve as valuable tools in stratifying PPI risk, offering either prognostic warning or reassurance to guide early post-TAVI management [7]. Anatomical characteristics of the left ventricular outflow tract, the coronary cusps, and the membranous septum—each subject to interindividual variability—also contribute to PPI risk, particularly when considered alongside procedural variables such as implantation technique and depth. In a study by Iacovelli *et al*. [8], involving 86 patients, each additional millimeter of implantation depth toward the left ventricular outflow tract was associated with a 1.41-fold increase in the likelihood of requiring PPI. The 2021 European Society of Cardiology (ESC) guidelines on cardiac pacing and cardiac resynchronization therapy, along with multiple expert consensus documents, have proposed structured algorithms for PPI decision-making following TAVI. However, these recommendations have yet to be validated by large-scale randomized trials or robust observational datasets. Furthermore, the heterogeneity of conduction disturbances contributes to clinical uncertainty, as there is currently no consensus on optimal management strategies [9, 10]. Another critical consideration is the potential for recovery of the conduction system, as certain conduction abnormalities may regress over time. This possibility supports the rationale for a watchful waiting approach in selected cases, particularly when early post-procedural conduction abnormalities are not definitive.

**Fig. 1.**
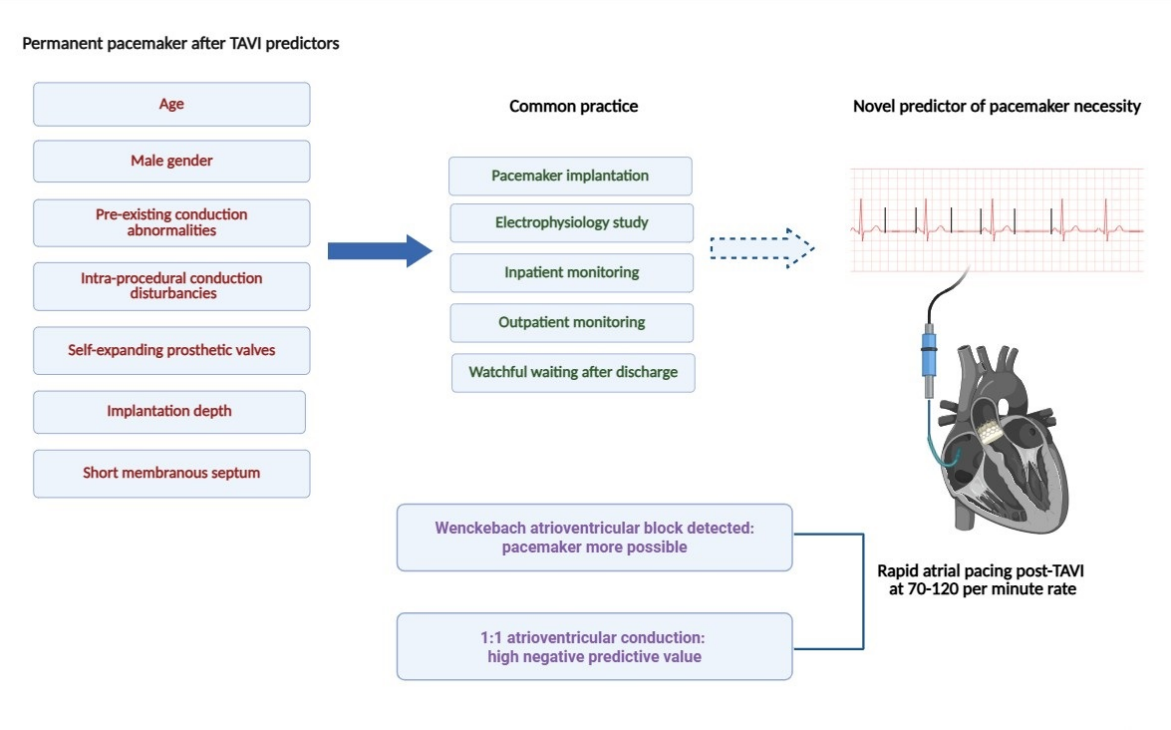
**Central illustration. Graphical illustration of rapid atrial pacing as a predictive tool for post-TAVI pacemaker implantation**. TAVI, transcatheter aortic valve implantation.

Electrophysiological testing to assess the risk or necessity of PPI following TAVI is supported by current guidelines and expert consensus statements. As a threshold-based approach, it offers a structured and objective means of risk stratification. While individualized clinical judgment remains paramount, predefined electrophysiological cut-offs can enhance decision-making confidence and facilitate standardized care. His-ventricular (HV) interval measurements have been evaluated in various clinical studies. The 2021 ESC guidelines recommend a threshold of 70 ms, whereas an observational study identified 55 ms as the cut-off in patients presenting with left bundle branch block [11]. Although HV interval assessment holds promise, its broader adoption is limited by several factors: a lack of consensus on definitive cut-offs, the limited availability of electrophysiological testing equipment in catheterization laboratories, and the general unfamiliarity of TAVI operators with these procedures. Furthermore, conduction disturbances following TAVI are often dynamic and may evolve over time. High-degree atrioventricular block can present in a delayed fashion, emerging hours to days after the procedure, or may resolve spontaneously as post-procedural edema and inflammation of the tissue adjacent to the aortic valve subside [12]. This variability further complicates the immediate post-TAVI decision-making process and underscores the need for ongoing monitoring and refined predictive tools (Fig. 1).

**Fig. 2.**
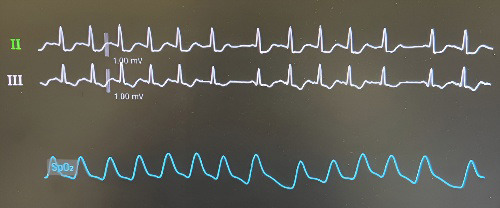
**Electrocardiographic recording demonstrating Wenckebach atrioventricular block induction during rapid atrial pacing**.

A promising avenue for risk stratification of AV conduction disturbances following TAVI may lie in the simple and widely applicable assessment of AV conduction using rapid atrial pacing via a temporary pacemaker lead (Fig. 2). In a study by Krishnaswamy *et al*. [13], rapid atrial pacing was performed post-TAVI at rates ranging from 70 to 120 beats per minute in 284 patients. The investigators found that the absence of pacing-induced Wenckebach-type AV block was associated with a very low likelihood of subsequent PPI [13]. In contrast, a more recent study by Tan *et al*. [14], involving 253 patients, reported that pacing-induced Wenckebach—whether observed before or after TAVI—did not reliably predict the need for PPI. Interpretation of these divergent findings requires caution. All patients in the study by Tan *et al*. [14] received balloon-expandable valves, whereas 75.7% of patients in the Krishnaswamy *et al*. [13] cohort did so, potentially accounting for some variability in outcomes. Moreover, the overall PPI rate was lower in the Tan *et al*. [14] study, and post-TAVI pacing tests were not performed in cases with pre-existing AV conduction disturbances (Table 1, Ref. [13, 14]). Notably, the incidence of new left bundle branch block (LBBB) was significantly higher among patients with a positive rapid atrial pacing test (21.3% vs. 9%, *p* < 0.007). Although these conflicting results do not yield definitive guidance, they offer important insights into the potential utility of rapid atrial pacing as a diagnostic tool. The test is time-efficient, low-cost, reproducible, and does not significantly prolong procedural duration. Given that the majority of TAVI procedures involve the placement of a temporary right ventricular pacemaker lead, transitioning to an atrial position post-implantation requires only a minor adjustment. Furthermore, comparing the Wenckebach cycle length before and after TAVI may help identify intraprocedural changes in conduction. While the optimal pacing threshold with strong negative predictive value has yet to be firmly established, current evidence provides a valuable foundation for future investigation into standardized conduction assessment strategies post-TAVI.

**Table 1.** **Summarized data of the recent studies investigating rapid atrial pacing post-TAVI**.

	Krishnaswamy *et al*. [13], 2020			Tan e*t al*. [14], 2023		
	RAP-induced Wenckenbach	No RAP-induced Wenckebach	*p*-value	RAP-induced Wenckenbach	No RAP-induced Wenckebach	*p*-value
Duration of enrollment (months)	13			16		
N	130	154		75	178	
Age (years)	82 (76.2–86)	81 (74.5–84.9)	0.404	80.0 ± 8.9	77 ± 9.4	0.02
Female (%)	45.8	50.3	0.108	40	47.5	0.26
Valves implanted	Sapien 3, CoreValve, Evolut–R, Lotus, Direct Flow			Sapien 3, Sapien 3 Ultra		
New persistent 1st degree AVB block	11.5	2.6		8	3.4	0.18
New persistent LBBB	9.2	9.7		21.3	9	**0.007**
Mortality (%)	0.8	0	0.458	1.3	1.1	1
PPI (%)	13.1	1.3	< **0.001**	13.3	8.4	0.23
Length of stay (days)	2.35 (1.7–5)	2.4 (1.7–4.7)	0.960	2.7 ± 4.5	2.3 ± 3.9	0.37
Follow-up length (days)	30			30		

AVB, atrioventricular block; LBBB, left bundle branch block; PPI, permanent pacemaker implantation; RAP, right atrial pacing; TAVI, transcatheter aortic valve implantation. Values are median (IQR), %, or mean ± SD. Statistically significant *p* values are highlighted with bold.

## 3. Discussion

We propose that rapid atrial pacing represents a promising strategy for risk stratification in patients undergoing TAVI and advocate its integration to inform decisions regarding PPI. Although it introduces an additional step to an already complex procedure, rapid atrial pacing enhances the clinician’s diagnostic armamentarium. A predictive model incorporating established PPI risk factors, such as advanced age, pre-TAVI electrocardiographic findings, and prosthetic valve type, combined with post-TAVI rapid atrial pacing, may yield substantial prognostic value and warrants further investigation. Given that patient safety remains paramount, the addition of an additional central venous access to facilitate pacing may elevate the risk of vascular complications and conflict with the contemporary minimalist TAVI approach. Nonetheless, at our institutions, the medial cubital vein is routinely utilized as an access site for the pacemaker lead, minimizing procedural complexity and associated risks.

## 4. Conclusion

Identifying and validating a reliable predictive tool is imperative, given the persistently high rates of PPI and the ongoing challenge of preventing delayed high-degree CD after TAVI. Larger, well-designed studies are required to determine the optimal pacing threshold and to rigorously evaluate the clinical utility of rapid atrial pacing. An upcoming observational study (NCT06189976) is anticipated to provide critical insights into these issues.

## Abbreviations

AV, atrioventricular; CD, conduction disturbances; ECG, electrocardiogram; ESC, European Society of Cardiology; PPI, permanent pacemaker implantation; TAVI, transcatheter aortic valve implantation.
